# “Drying effect” of fructus aurantii components and the mechanism of action based on network pharmacology and *in vitro* pharmacodynamic validation

**DOI:** 10.3389/fphar.2023.1114010

**Published:** 2023-03-08

**Authors:** Jing Zhu, Yi Luo, Hengli Tong, Lingyun Zhong, Qianfeng Gong, Yaqi Wang, Ming Yang, Qing Song

**Affiliations:** ^1^ Pharmacy College, Jiangxi University of Traditional Chinese Medicine, Nanchang, China; ^2^ Ultrasound Diagnosis Department of Jiangxi Traditional Chinese Medicine Hospital, Nanchang, China

**Keywords:** fructus aurantii, dryness, action mechanism, active components, systems pharmacology

## Abstract

**Background:** Fructus aurantii (FA) is the dried, unripe fruit of the plant *Citrus aurantium* L. and its cultivated varieties. We investigated the drying effect of FA components and how this drying affect is achieved.

**Methods:** We employed systems pharmacology to predict the components and targets of FA that produce its drying effect. These predictions were verified by computer simulation and animal experiments. In the latter, we measured the bodyweight, water consumption, urine output, fecal water content, rate of salivary secretion, and cross-sectional area of the long axis of the submandibular gland of mice. Immunohistochemistry was used to measure expression of aquaporin (AQP)5 in the submandibular gland, AQP2 in the kidney, and AQP3 in the colon. ELISA kits were used to measure the horizontal variation of cyclic adenosine monsophosphate (cAMP), cyclic guanosine monophosphate (cGMP) and interferon-γ.

**Results:** Sixty-seven potentially active components of FA were screened out. FA could produce a drying effect after regulating 214 targets through 66 active components. A total of 870 gene ontology (GO) terms and 153 signaling pathways were identified. The hypoxia inducible factor-1 signaling pathway, phosphoinositide 3-kinase-protein kinase B (PI3K-AKT) signaling pathway, calcium signaling pathway, and Ras signaling pathway may have important roles in the drying effect of FA. Four components of FA were identified: sinensetin, tangeretin, 5-demethylnobiletin and chrysin. These four components could increase the serum level of interferon-γ and ratio of cyclic adenosine monophosphate:cyclic guanosine monophosphate in mice, and affect their water consumption, urine output, fecal water content and rate of salivary secretion.

**Conclusion:** Four components of FA (tangeretin, sinensetin, chrysin, 5-Demethylmobiletin) were closely related to the Janus kinase-signal transducer and activator of transcription-3 (JAK-STAT3), PI3K-AKT, and the other signaling pathways. They can regulate the protein expression of JAK2, STAT3, PI3K, lymphocyte cell-specific protein-tyrosine kinase, vascular endothelial growth factor A, and protein kinase B1, affect water metabolism in the body and, finally, result in a drying effect.

## 1 Introduction

The quality of extracts of traditional Chinese medicine (TCM) formulations is important to determine if such formulations can have a clinical effect. Many Chinese botanical drugs have been reported to have pharmacological and biological (e.g., antioxidant, anti-inflammatory, antibacterial, antiviral, anti-tumor) activities. (Nikzad-Langerodi et al., 2017; Uysal et al., 2017).

The side effect of dryness affects the efficacy of TCM formulations. On the one hand, physicians can use the dryness of a TCM formulation to dispel dampness and treat dampness syndrome. On the other hand, the dryness of a TCM formulation damages body-fluid metabolism, resulting in a dry mouth and throat (Wang et al., 2014). The properties or toxicity of a TCM formulation can be altered by processing, such as frying, stir-frying, calcining, steaming, boiling, blanching, or water milling (Ye et al., 2020; Cao et al., 2022).

Fructus aurantii (FA) is the dry immature fruit of *Citrus aurantium* L. and its cultivated varieties. It can promote Qi circulation to alleviate the middle energizer. The dryness of FA is usually regarded as its adverse effect in the clinical setting. With regard to the drying effect of raw FA, *Shennong’s Herbal Classic* says that “those who cough with Yin deficiency and inflammation will be in danger after taking it”. Modern experimental research shows that raw FA will destroy the metabolism of body fluids (Zhu et al., 2020).

Sjögren’s syndrome (SS; also known as “autoimmune exocrine adenosis” or “autoimmune exocrine supraglandular dermatitis”) is characterized mainly by histology and functional changing in exocrine glands (e.g., lacrimal glands, submandibular gland, parotid glands, pancreas) (Chatzis et al., 2021). The clinical manifestations of SS are dry mouthand dry eyes, etc.

“Systems pharmacology” can be used to screen out the effective substances of a TCM formulation and analyze its mechanism of action by constructing a multi-level “component–target–pathway” network (Hopkins, 2008; Li et al., 2014; Zhang et al., 2019). This strategy enables systematic determination of the effect and mechanism of the drug action for treating complex diseases at molecular, cellular, tissue, and biological levels (Yang et al., 2019).

**FIGURE 1:**
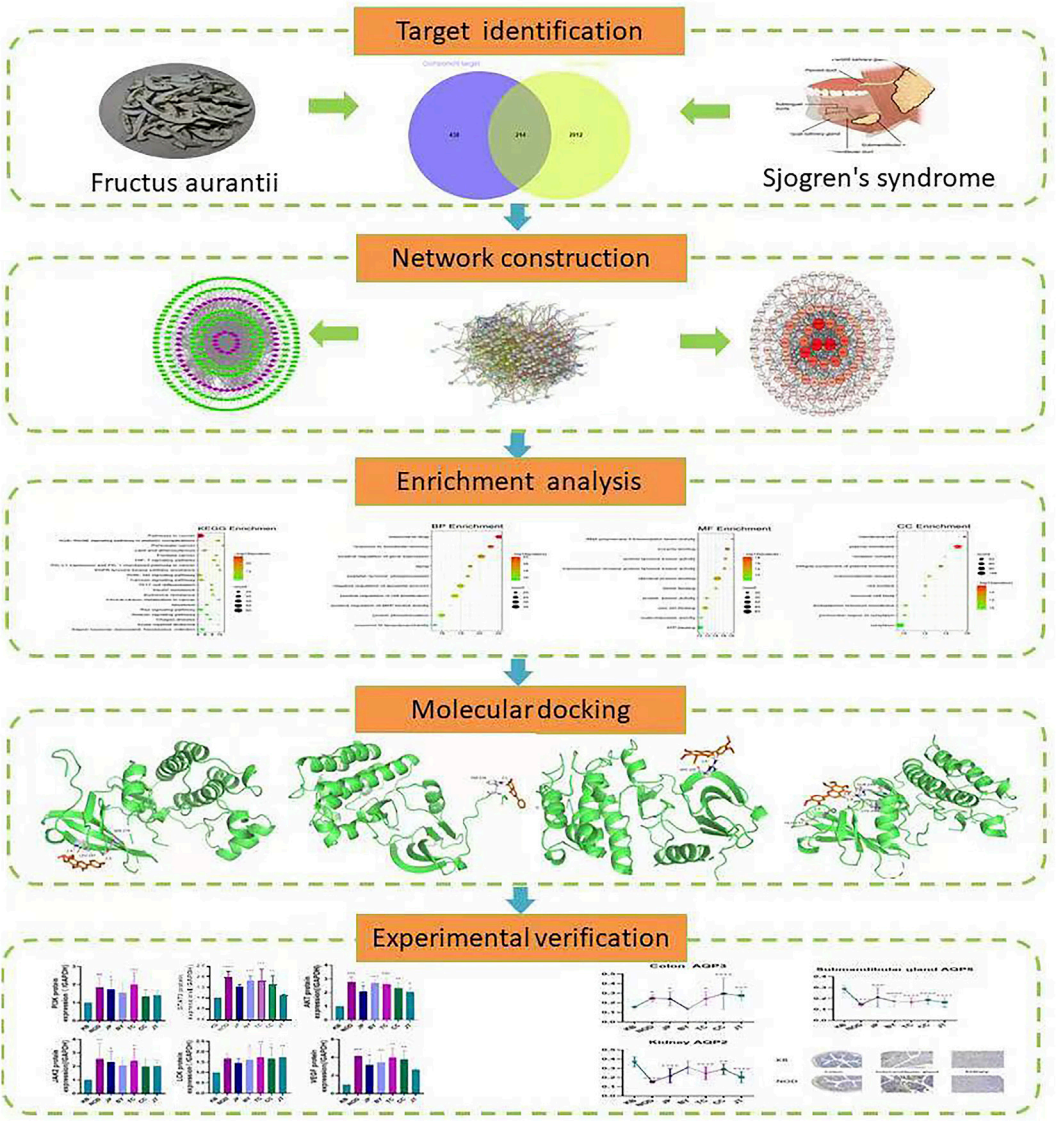
Systems pharmacology-based strategy to study the drying effect of components of Fructus Aurantii and the mechanism of action.

In this situation, Systems Pharmacology was used to analyze the active compounds, drug targets, and key signaling pathways of FA to ascertain how the drying effect worked (Figure 1). We conducted ultrasound examination at the submandibular glands of mice. Combining the traditional evaluation indicators (e.g., salivary flow rate, water consumption, urine volume), we sought to establish an evaluation method of the drying effect of FA. In this way, we wished to lay a foundation for the study of the drying effect of FA and reveal its mechanism of action.

## 2 Materials and methods

### 2.1 Ethical approval of the study protocol

The study protocol was approved (JZLLSC20220813) by the Research Ethics Committee of Jiangxi University of Traditional Chinese Medicine (Nanchang, China). Potentially identifiable human images or data are not presented in this study.

### 2.2 Chemicals, agents, and materials

A decolorization shaker (ZD-9560) was purchased from Jiangsu Shenglan Instrument Manufacturing (Jiangsu, China). An electrophoresis instrument (JY300HC), vertical electrophoresis tank (JY-SCZ2+), and western blotting system transfer electrophoresis tank (JY-ZY5) were obtained from Beijing Junyi Huaxin Technology (Beijing, China). A desktop high-speed freezing centrifuge (TGL-16M) and chemiluminescence imaging device (chemiscope Mini 3300) were sourced from Shanghai Qinxiang Scientific Instruments (Shanghai, China).

A bicinchoninic-acid protein quantification kit was obtained from Beijing Solarbio Technology (Beijing, China). Polyvinylidene-fluoride membranes and an electrochemiluminescence kit were purchased from Millipore (Burlington, MA, United States).

Antibodies against glyceraldehyde 3-phosphate dehydrogenase, signal transducer and activator of transcription (STAT)3, and protein kinase B (AKT) were obtained from Abcam (Cambridge, United Kingdom). Phosphoinositide 3-kinase (PI3 kinase) p85 rabbit monoclonal antibody was purchased from Cell Signaling Technology (Danvers, MA, United States). Preserved protein ladders were from Fermentas (Burlington, ON, United States). Lymphocyte cell-specific protein-tyrosine kinase (Lck) polyclonal antibody was sourced from Proteintech (Chicago, IL, United States). Rabbit anti-vascular endothelial growth factor (VEGF) antibody was obtained from Beijing Boarsen Biotechnology (Beijing, China). Antibody against Janus kinase (JAK)2 was purchased from Hangzhou Hua’an Biotechnology (Hangzhou, China). Antibodies against horseradish peroxidase-conjugated goat anti-rabbit immunoglobulin-G (H + L) and goat anti-mouse immunoglobulin-G (H + L) were sourced from Beijing TDY Biotechnology (Beijing, China).

As reference standards, four compounds with purity ≥98% were used. They were sinensetin (batch number: wkq21060106), tangeretin (wkq21042202), 5-demethylnobiletin (wkq21052610), and chrysin (wkq21042612), which were purchased from Sichuan Vicky Biotechnology (Sichuan, China).

### 2.3 Collection of FA components and prediction of candidate compounds

Using “Fructus aurantii” as the keyword, we searched the China National Knowledge Infrastructure, VIP, and Web of Science databases to discover the ingredients contained in raw pieces of FA. Then, by comparison with the ingredients included in Traditional Chinese Medicine Systems Pharmacology (TCMSP) database and Traditional Chinese Medicine Integrated Database (TCMID), the information of each ingredient was summarized after removing duplicate items (Ji et al., 2006; Ru et al., 2014).

Next, we used the Lipinski’s Rule of Five (Lipinski et al., 2012). That is, if a FA component was subject to certain provisions of certain parameters, it could be identified as a potentially effective component. These provisions werehydrogen-bond donor (number of hydrogen atoms attached to oxygen and nitrogen atoms) ≤5; Relative molecular mass ≤500; Partition coefficient of fat:water miLogP ≤5; Hydrogen-bond acceptor (number of oxygen and nitrogen atoms) ≤10; Meeting at least two of four filters (Ghose (Amgen), Veber (GSK), Egan (Pharmacia) and Muegge (Bayer); Gastrointestinal Absorption Index was “high” (Sravika et al., 2021; Zheng et al., 2022).

### 2.4 Identification of the related targets of FA components

Collected ingredient was searched for in PubChem (www.ncbi.nlm.nih.gov/pubmed) and ChemicalBook (www.chemicalbook.com/). These platforms can be used to distinguish the molecular structure of ingredients (Dashti et al., 2019). The structure data file (SDF) of ingredients was downloaded from PubChem. All the targets of the compounds in FA were collected from SwissTargetPredition (www.swisstargetprediction.ch/). After removing redundant information, 66 components in FA and 652 known targets associated with them were obtained. Then, through the UniProt database (www.uniprot.org/), we undertook conversion to the gene name (The uniProt consortium, 2021) for future use.

### 2.5 Acquisition of targets for SS

GeneCards (www.genecards.org/), DisGeNET (www.disgenet.org/) and Online Mendelian Inheritance in Man (www.omim.org/) were searched using the keywords “Sjögren syndrome”, “Sjogren’s syndrome”, “Sjogren’s disease”, and “sicca syndrome”. We deleted duplicates to obtain relevant targets (Stelzer et al., 2016; Yu et al., 2020).

### 2.6 Construction of protein-protein interaction (PPI) and compound-target networks

First, we intersected the obtained drug targets with the disease related target, then we obtained a Venn diagram of the intersecting targets. After that, we inputted the intersected targets into Search Tool for the Retrieval of Interacting Genes/Proteins (STRING) 11.5 (https://string-db.org/). STRING can be used to predict the interaction relationship between targets. We selected “multiple proteins” in the left functional area and set the species as *Homo sapiens*. We analyzed the interaction relationship between common targets, then imported the results into Cytoscape 3.7.1 (www.cytoscape.org/) to build the PPI network “compound-target” network. We used the “network analyzer” function to analyze the network parameters and visualize the results (Zhu and Hou, 2020; Li et al., 2021).

### 2.7 Enrichment analyses

To explore the biological process (BP), molecular function (MF), cellular component (CC), and related pathways associated with the drying effect of FA, the intersection targets obtained previously were enriched and analyzed using Database for Annotation, Visualization and Integrated Discovery (DAVID) 6.8 (https://david.ncifcrf.gov/home.jsp) (Cui et al., 2020; Zhang et al., 2020; Gegen et al., 2022). We selected *p* < 0.05 as the screening criterion. Analysis of enrichment of function and signaling pathways was conducted using the Gene Ontology (http://geneontology.org/) database and Kyoto Encyclopedia of Genes and Genomes (www.genome.jp/) database, respectively. The top-20 enriched genes were visualized using ImageGP (www.ehbio.com/ImageGP/). We studied the relationship between components, common targets, and pathways, and predicted how FA elicits its drying effect.

### 2.8 Expression of targets in organs

TCM theory emphasizes the holistic nature of treatment. Usually, TCM preparations treat diseases by regulating multiple organs to achieve a “peaceful” state (Li, 2016). We wished to characterize the relationship between the efficacy of FA and organs. Consequently, we imported the targets in the top-15 nodes of the PPI network into the biogps database (http://biogps.org/). The species we chose was “human”. We obtained protein-expression datasets of related targets in different organs, and generated organ-expression heatmaps of targets.

### 2.9 Computational validation of compound-target interactions

We used computer simulations to study active compounds and their targets. We selected the top-10 potentially active components in FA for molecular docking with the top-15 node targets in the PPI network. CB-dock (http://cao.labshare.cn/cb-dock/) is a docking tool for docking protein-ligand complexes. CB-dock can identify binding sites, calculate the location of docking centers, and automatically customize the size of docking-box according to ligands. After that, we ran AutoDock Vina (https://vina.scripps.edu/) (Liu et al., 2022a; Yang et al., 2022).

We obtained and downloaded the SDF of FA potential components from PubChem. The entry codes corresponding to the top-15 targets in the PPI network can be found in the Research Collaboration for Structural Bioinformatics in Protein Database (PDB; www.rcsb.org/). Then, we downloaded their PDB format. CB-dock uses curvature based cavity detection (curpocket) to predict the binding sites of target proteins. Then, the binding posture of the query ligand can be predicted using AutoDock Vina, which provides a guarantee for the accuracy of molecular docking. The more negative the docking score, the more complex is the interaction between the target and compound molecule.

### 2.10 Experimental validation

#### 2.10.1 Screening of the best ingredients and dose

Dosing for mice was calculated based on the doses of drugs used in humans and converted accordingly (Reagan-Shaw et al., 2008; Chinese Pharmacopoeia Commission, 2020). In pharmacological experiments, drugs are often prepared in a solution of a certain concentration and then administered in mL/kg body-weight. As long as the difference between the animal’s body-weight and standard body-weight is less than ±20%, the drug can be administered at the same dose.

The range in content of four components of FA from 10 locations was determined using raw pieces of FA from these 10 places. The content range (in μg/g) of the four components in 10 batches of raw FA was 9.27–283.84 for sinensetin, 36.01–610.83 for chrysin, 164.31–2994.21 for tangeretin, and 6.60–301.78 for 5-Demethylmobiletin (see the Supplementary Table for details). Then, according to the maximum content of each component, we calculated the amount of the four components in the maximum dose of FA prescribed in the *Chinese Pharmacopoeia* (10 g).

We performed equivalent dose switching in mice. That is, we multiplied the dose for a human (60 kg) per kilogram of body-weight by the conversion coefficient of a human and a mouse under standard weight (12.33) to obtain the dose of a mouse per kilogram of body-weight (Db) of standard body-weight (0.02 kg). The body-weight of an experimental mouse changes dynamically. Hence, the dose for a mouse with a non-standard body-weight (Db_1_) = Db·Sb (where Sb is the correction coefficient of a mouse with a non-standard body-weight) (Table 1).

**TABLE 1:** Calibration coefficients for animals of non-standard weight (S_b_).

B = W/W_标_	0.6	0.7	0.8	0.9	1.0	1.1	1.2	1.3	1.4
S_b_ = 1/B^1/3^	1.186	1.126	1.077	1.036	1.0	0.969	0.941	0.916	0.894

#### 2.10.2 Preparation of drugs

The FA used for the administration of the extract was purchased from Jiangxi Guhan Refined Chinese Herbal Pieces (Jiangxi, China). It was identified by Professor Zhang Jinlian of the Chinese Herbal Processing Discipline Group of Jiangxi University of Traditional Chinese Medicine (Jiangxi, China) as the dried immature fruit of the Rutaceae plant Citrus aurantium. First, we weighed an appropriate amount of raw FA. Then, we added 10-times the amount of water and boiled for 30 min. This action was repeated twice. Next, we combined the filtrate, decompressed and concentrated to weigh 1 g/ml of the drug solution. Lastly, we gavaged the drug according to the body-weight of mice.

In addition, considering the low efficiency and insufficient purity of the monomer components purified by our research team, the monomer compounds used in the monomer-component administration group were purchased from professional companies specializing in extracting single compounds from plant components.

The four components were flavonoids and had poor solubility in water. Hence, 0.1% dimethyl sulfoxide (DMSO) was added when preparing the solution for administration (Gilfanova et al., 2021; Hoyberghs et al., 2021). To ensure consistency, the same dose of DMSO solution was also added in physiologic (0.9%) saline.2.10.3Validation of pharmacodynamics sixty healthy, specific pathogen-free (SPF), female BALB/C mice (license number: SCXK(Yu) 2020-0005) were purchased from Henan Sikebasi Biotechnology (Henan, China). Ten female SPF non-obese mice suffering from diabetes mellitus (NOD/LtJ) were purchased from SBF (Beijing) Biotechnology (license number: SCXK(Beijing) 2019-0010) in Beijing, China.

After 1 week of adaptive feeding, 60 BALB/C mice were divided into six groups of ten: control; extract administration; sinensetin; tangeretin; 5-demethylnobiletin; chrysin. In addition, 10 female NOD/Ltj mice were set as the model (NOD) group. Mice in each administration group were administered the agent by gavage at 10-times the equivalent dose for 5 weeks. The control group and NOD group were given an identical volume of physiologic (0.9%) saline.

The body-weight and water consumption of mice were recorded every day. At days 0, 7, 14, 21, 28, and 35 of administration, mice were placed in the corresponding metabolic cages in groups. The total urine output of mice in each group was measured at 12 h. Feces were collected within 12 h, and foreign matter and other impurities removed. The moisture of the dejecta in each group was measured. The rate of salivary secretion of mice in each group was measured 0, 15, 18, 21, 24, and 27 days after administration. Thirty-two days after 32 administration, the submandibular glands of mice were examined using a high-resolution small-animal ultrasound imaging system (VEVO 2100; VisualSonics, Toronto, Canada).

After 35 days of administration, mice in each group were fasted for 12 h without water. Blood was taken from the retro-orbital sinus. Serum was collected by centrifugation (Relative centrifugal force (RCF) = 684.775 g, 10 min, 4°C). The level of interferon (IFN)-γ, cyclic adenosine monophosphate (cAMP), and cyclic guanosine monophosphate (cGMP) was determined by enzyme-linked immunosorbent assays (ELISAs). Expression of VEGF, JAK2, phosphoinositide 3-kinase-protein kinase B (PI3K), STAT3, Lck, and AKT in mouse colons was determined by immunoblotting.

### 2.11 Statistical analyses

Measurement data are expressed as the mean ± standard deviation. Prism 8.0.2 (GraphPad, San Diego, CA, United States) was used for one-way ANOVA to determine the significance of results. *p* < 0.05 was considered significant. All experiments were repeated at least three times.

## 3 Results

### 3.1 Collection and screening of the raw ingredients of FA

According to the screening criteria described in Section 2.3 (Lipinski’s Rule of Five and four filters), 43 compounds met the screening requirements, and could be used as candidates for screening the active components of FA.

Even though some components do not meet the screening criteria and their pharmacokinetic values are relatively low, several studies have reported their biological activities (Dagur et al., 2022). They also need to be included in a library of candidate components for screening the active compounds of FA to investigate this issue more fully. Twenty-four components (e.g., naringin, neohesperidin, naringin, rutin, hesperidin, limonene) did not meet the screening criteria stated above, but they were retained as active components. Finally, 67 compounds were selected as candidate active ingredients of FA (Table 2).

**TABLE 2:** Candidate active ingredients of Fructus Aurantii.

NO	Name of molecular	Encoded	MW	CAS
1	2-acetyloxyethyl (trimethyl)azanium	M1	C_7_H_16_NO_2_^+^	51-84-3
2	2-hydroxybenzoic acid	M2	C_7_H_6_O_3_	69-72-7
3	5,7-dihydroxy-2-(4-hydroxyphenyl)-2,3-dihydrochromen-4-one	M5	C_15_H_12_O_5_	67604-48-2
4	Palmitic Acid	M7	C_16_H_32_O_2_	57-10-3
5	4-(2-amino-1-hydroxyethyl)phenol	M11	C_8_H_11_NO_2_	104-14-3
6	Terbutaline	M13	C_12_H_19_NO_3_	23031-25-6
7	4-[1-hydroxy-2-(methylamino)ethyl]phenol	M18	C_9_H_13_NO_2_	94-07-5
8	7-methoxy-8-(3-methylbut-2-enyl)chromen-2-one	M25	C_15_H_16_O_3_	484-12-8
9	2-methyl-5-propan-2-ylphenol	M26	C_10_H_14_O	499-75-2
10	3-hydroxy-2-phenylchromen-4-one	M28	C_15_H_10_O_3_	577-85-5
11	Tangeretin	M32	C_20_H_20_O_7_	481-53-8
12	Hesperetin	M35	C_16_H_14_O_6_	520-33-2
13	Nobiletin	M36	C_21_H_22_O_8_	478-01-3
14	Tetramethylscutellarein	M37	C_19_H_18_O_6_	1168-42-9
15	Sinensetin	M39	C_20_H_20_O_7_	2306-27-6
16	5-Demethylnobiletin	M41	C_26_H_30_O_8_	2174-59-6
17	Naringenin	M42	C_15_H_12_O_5_	480-41-1
18	4-[(1R)-2-amino-1-hydroxyethyl]phenol	M43	C_8_H_11_NO_2_	876-04-0
19	Fumaric Acid	M47	C_4_H_4_O_4_	110-17-8
20	Ferulic Acid	M49	C_10_H_10_O_4_	537-98-4
21	Coumaric Acid	M50	C_9_H_8_O_3_	501-98-4
22	(2R)-5,7-dihydroxy-2-(3-hydroxy-4-methoxyphenyl)-2,3-dihydrochromen-4-one	M55	C_16_H_14_O_6_	69097-99-0
23	4-[(1R)-1-hydroxy-2-(methylamino)ethyl]phenol	M57	C_9_H_13_NO_2_	614-35-7
24	Auraptene	M61	C_19_H_22_O_3_	495-02-3
25	N-[2-(4-methoxyphenyl)ethyl]benzamide	M63	C_16_H_17_NO_2_	3278-19-1
26	4-azaniumylbenzenesulfonate	M64	C_6_H_7_NO_3_S	121-57-3
27	7-hydroxychromen-2-one	M68	C_9_H_6_O_3_	93-35-6
28	Kaempferide	M69	C_16_H_12_O_6_	491-54-3
29	5-methyl-2-propan-2-ylphenol	M83	C_10_H_14_O	89-83-8
30	pentadecanoic acid	M138	C_15_H_30_O_2_	1002-84-2
31	3,3′,4′,5,6,7,8-Heptamethoxyflavone	M147	C_22_H_24_O_9_	1178-24-1
32	Isosakuranetin	M148	C_16_H_14_O_5_	480-43-3
33	5,6,7,3′,4′,5′-Hexamethoxyflavone	M150	C_21_H_22_O_8_	29043-07-0
34	Eriodictyol	M151	C_15_H_12_O_6_	552-58-9
35	Acacetin	M155	C_16_H_12_O_5_	480-44-4
36	Scopoletin	M156	C_10_H_8_O_4_	92-61-5
37	Chrysin	M157	C_15_H_10_O_4_	480-40-0
38	Diosmetin	M158	C_16_H_12_O_6_	520-34-3
39	(1^13^C)dodecanoic acid	M159	C_12_H_24_O_2_	93639-08-8
40	Quercetin	M65	C_15_H_10_O_7_	117-39-5
41	Apigenin	M66	C_15_H_10_O_5_	520-36-5
42	Kaempferol	M67	C_15_H_10_O_6_	520-18-3
43	Robinetin	M70	C_15_H_10_O_7_	490-31-3
44	Naringin	M162	C_27_H_32_O_14_	10236-47-2
45	Neohesperidin	M163	C_28_H_34_O_15_	13241-33-3
46	Narirutin	M165	C_27_H_32_O_14_	14259-46-2
47	(-)-Limonene	M164	C_10_H_16_	5989-54-8
48	Limonene	M161	C_10_H_16_	5989-27-5
49	Hesperidin	M160	C_28_H_34_O_15_	520-26-3
50	alpha-Farnesene	M166	C_15_H_24_	502-61-4
51	beta-Elemene	M167	C_15_H_24_	515-13-9
52	gamma-Terpinene	M168	C_10_H_16_	99-85-4
53	O-Cymene	M169	C_10_H_14_	527-84-4
54	M-Cymene	M170	C_10_H_14_	535-77-3
55	(-)-alpha-Cubebene	M171	C_15_H_24_	17699-14-8
56	Neoeriocitrin	M172	C_27_H_32_O_15_	13241-32-2
57	(-)-alpha-Pinene	M173	C_10_H_16_	7785-26-4
58	(+)-alpha-Pinene	M174	C_10_H_16_	7785-70-8
59	alpha-Terpinene	M175	C_10_H_16_	99-86-5
60	Naringin 4′-glucoside	M176	C_33_H_42_O_19_	17257-21-5
61	Hesperetin 5-O-glucoside	M177	C_22_H_24_O_11_	69651-80-5
62	Merancin hydrate	M178	C_15_H_18_O_5_	5875-49-0
63	Meranzin	M180	C_15_H_16_O_4_	23971-42-8
64	Quercimeritrin	M181	C_21_H_20_O_12_	491-50-9
65	Naringenin chalcone	M182	C_15_H_12_O_5_	73692-50-9
66	Isomeranzin	M183	C15H16O4	1088-17-1
67	Xanthotoxol	M141	C_11_H_6_O_4_	2009-24-7

### 3.2 Identification of the related targets of FA constituents

All the targets of the compounds in FA were collected from SwissTargetPredition and TCMSP databases. After removing redundant information, 67 components in FA and 652 known targets associated with these components were obtained (Supplementary Table).

### 3.3 Acquisition of the known therapeutic targets for SS

After collecting and removing redundant items from GeneCards, DisGeNET, and OMIM databases, 3126 known targets of SS treatment were collected (Supplementary Table).

### 3.4 Analyses of the compound–target network

**FIGURE 2:**
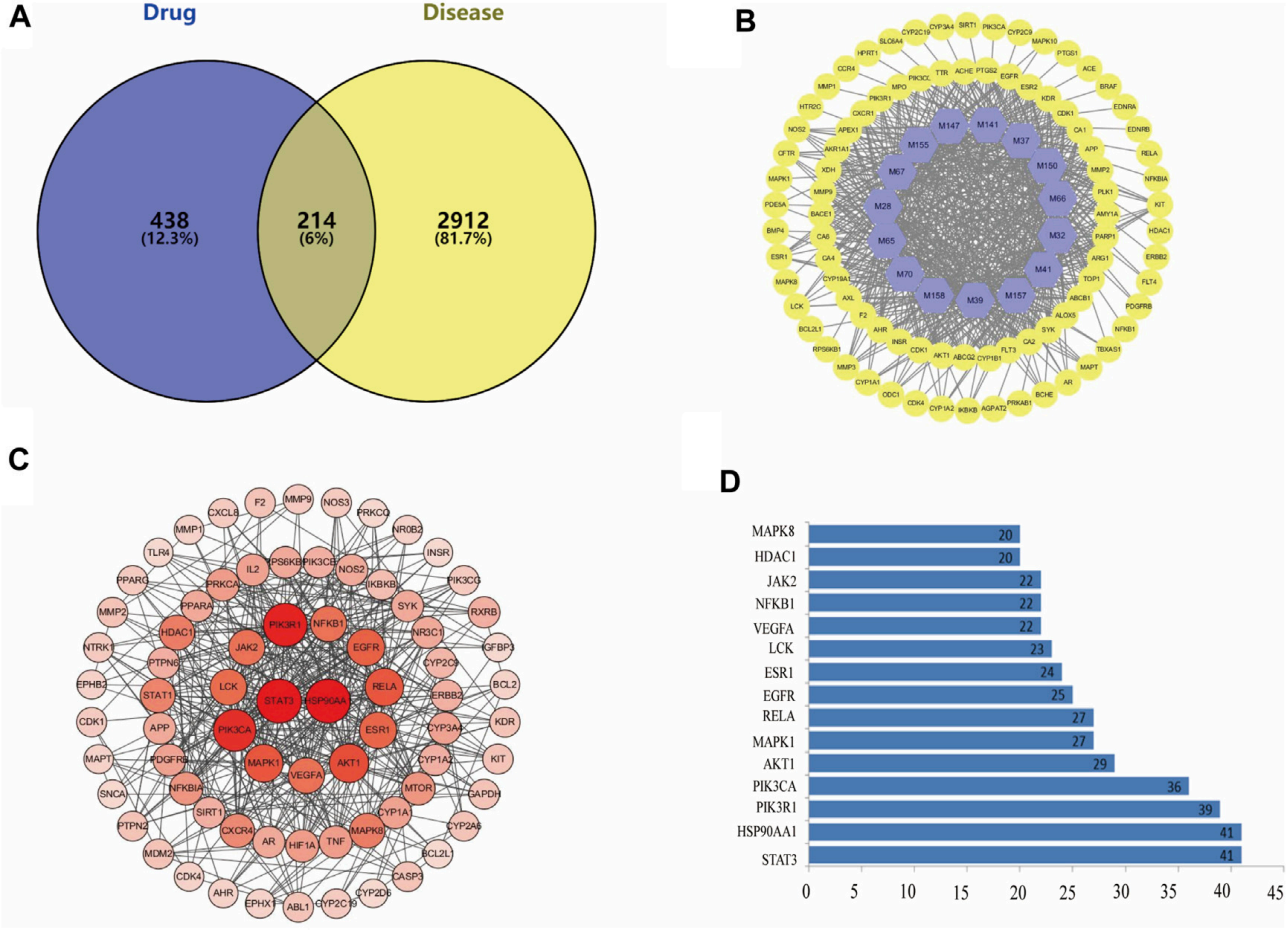
**(A)** There are 214 overlapping targets between disease and drug. **(B)** C-T network. The 86 warm yellow nodes indicate the potential active ingredients of dryness effect. The 15 light blue nodes indicate coincident targets between disease and drug. The edges denote that nodes can interact with each other. **(C)** Details of the PPI Network. According to the degree value of each target, the larger the degree value, the redder the color, and the larger the circle. **(D)** Bar plot of the PPI network. The x-axis represents the number of neighboring proteins of the target protein. The y-axis represents the target protein.

There were 214 overlaps of 3126 targets for disease and 652 targets for the drug (Figure 2A). That is, there could be 214 key targets for FA during SS treatment. The 214 overlapping targets are detailed in Supplementary Table.

To elucidate how FA elicits a dryness effect symptoms, we constructed a Compound-Target network. Figure 2B shows the 15 potential active components which may act on 86 targets to exert a dryness effect. The 15 light blue nodes represent active components in FA; The 86 warm yellow nodes represent potential targets which acted by the components in FA; Edges indicate that nodes could influence each other.

### 3.5 Analyses of the PPI network

We constructed a PPI network consisting of 65 nodes and 443 edges. This PPI network was based on the premise that proteins interact with each other more than would be expected for a random proteome of similar size extracted from the genome. This enrichment suggests that the proteins are at least partially linked biologically as a group (Figure 2C). Based on the PPI network’s “Betweenness unDir” and other analysis parameters, we screened 32 potential core targets related to dryness (Supplementary Table).

We identified the top-15 proteins in the PPI network. STAT3 and heat shock protein (HSP)90AA1 may be associated with 41 other proteins (Figure 2D). Phosphoinositide-3-kinase regulatory subunit 1 (PIK3R1) was related to 39 other proteins. PIK3CA was associated with 36 other proteins. These results suggested that these four proteins would be the focus of our study on PPIs. Fucosyltransferase (FUT)7 and serine/threonine-protein kinase TNNI3K were not associated with other proteins in this PPI network, implying that they were less important.

### 3.6 Enrichment analyses

**FIGURE 3:**
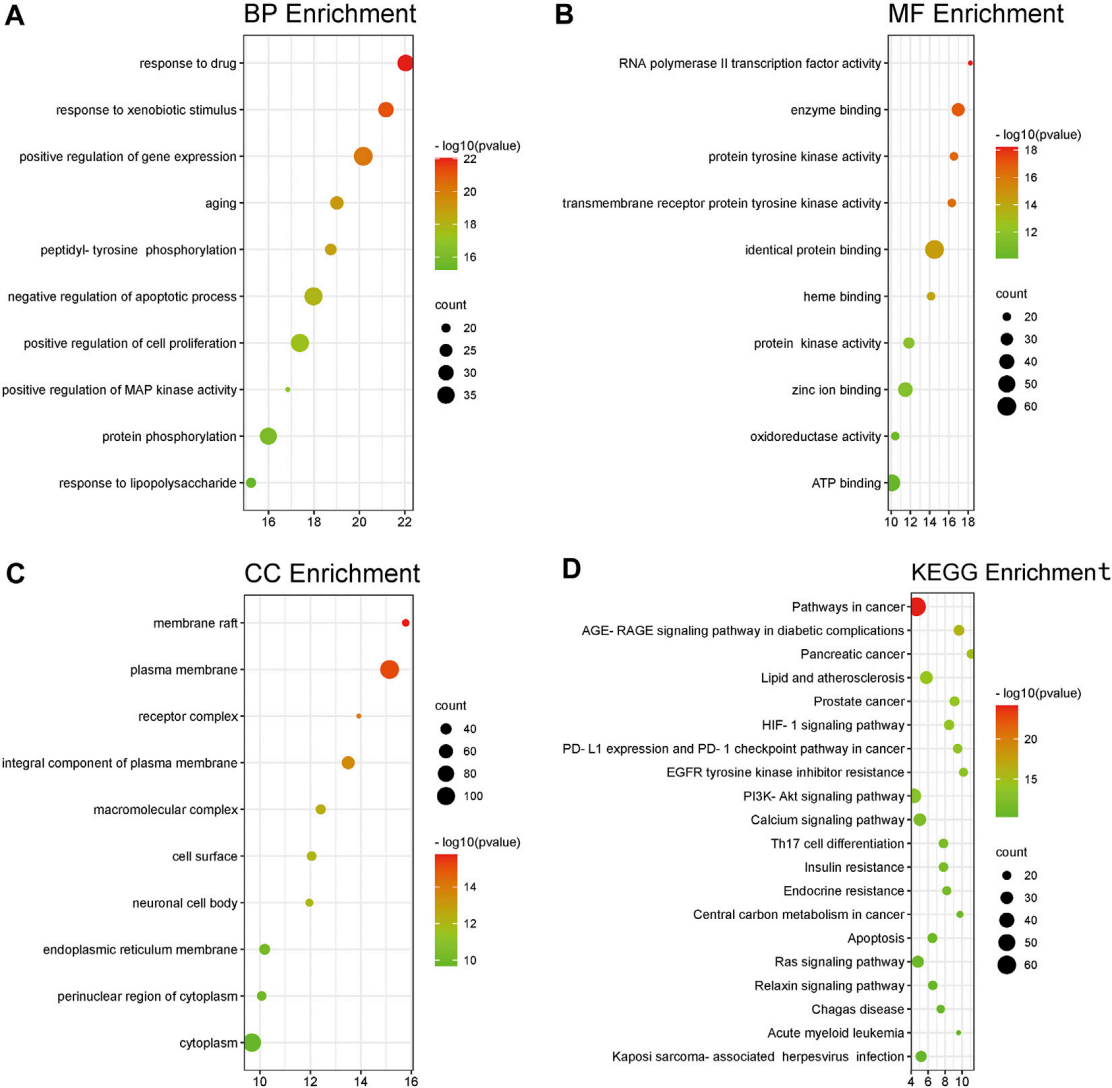
**(A–C)** Analyses of 214 targets associated with Sjögren’s syndrome using the GO database. The x-axis represents significant enrichment of these term counts. The y-axis represents the category of “biological process”, “cellular component” and “molecular function” in the GO of target genes (*p* < 0.05, top10). **(D)** Pathway-enrichment analyses using the KEGG database. The x-axis represents the count of targets in each pathway; the y-axis represents the primary pathway (*p* < 0.01, top20).

First, we carried out enrichment analyses using DAVID using GO terms to elucidate relevant BP. The y-axis represents GO terms. The x-axis indicates the number of targets enriched in this term. The redder the color, the smaller the *p*-value, the higher is the credibility and importance. In contrast, the greener the color, the greater is the value of p_adjusted_. The size of the circle represents the number of genes included in the corresponding enrichment entry. The larger the circle area, the more genes that are included. The top-10 terms of BP, CC, and MF were screened (Figures 3A–C).

Details of pathway-enrichment analyses using DAVID are provided in Supplementary Table. The main BP were “response to a drug” (GO:0042493), “response to a xenobiotic stimulus” (GO: 0009410), “positive regulation of gene expression” (GO:0010628), “aging” (GO:0007568), and “peptidyl-tyrosine phosphorylation” (GO:0018108). The main CC were “membrane raft” (GO:0045121), “plasma membrane” (GO:0005886), “receptor complex” (GO:0043235), “integral component of plasma membrane” (GO:0005887), and “macromolecular complex” (GO:0032991). The main MF were “RNA polymerase II transcription factor activity (GO:0004879)”, “ligand-activated sequence-specific DNA binding” (GO:0004879), “enzyme binding” (GO:0019899), “protein tyrosine kinase activity” (GO:0004713), “transmembrane receptor protein tyrosine kinase activity” (GO:0004714), and “identical protein binding” (GO:0042802).

The y-axis of Figure 3D represents a signaling pathway. The x-axis indicates the enrichment multiple of this signaling pathway. A total of 214 overlapping targets were mapped to 154 signaling pathways. For a more intuitive presentation, a bubble diagram can be drawn by intercepting the position of top-20 signaling pathways from small to large according to the *p*-value. The details of signaling-pathway enrichment based on the KEGG database are shown in Supplementary Table.

Analyses of signaling-pathway enrichment using DAVID indicated that the raw ingredients of FA leading to a drying effect were based on “immune response”, “inflammation”, and “tumor or cancer”. Two-hundred and fourteen overlapping targets were closely related to “pathways in cancer (hsa05200), “HIF-1 signaling pathway (hsa04066)”, “PI3K-AKT signaling pathway” (hsa04151), “Th17 cell differentiation” (hsa04659), “calcium signaling pathway” (hsa04020), and “Ras signaling pathway” (hsa04014) (Figure 3D). These signaling pathways may be the key ones leading to the drying effect of FA. This type of analysis provides a new method to elucidate how the various components of FA elicit a drying effect.

### 3.7 Expression of targets in different organs

**FIGURE 4:**
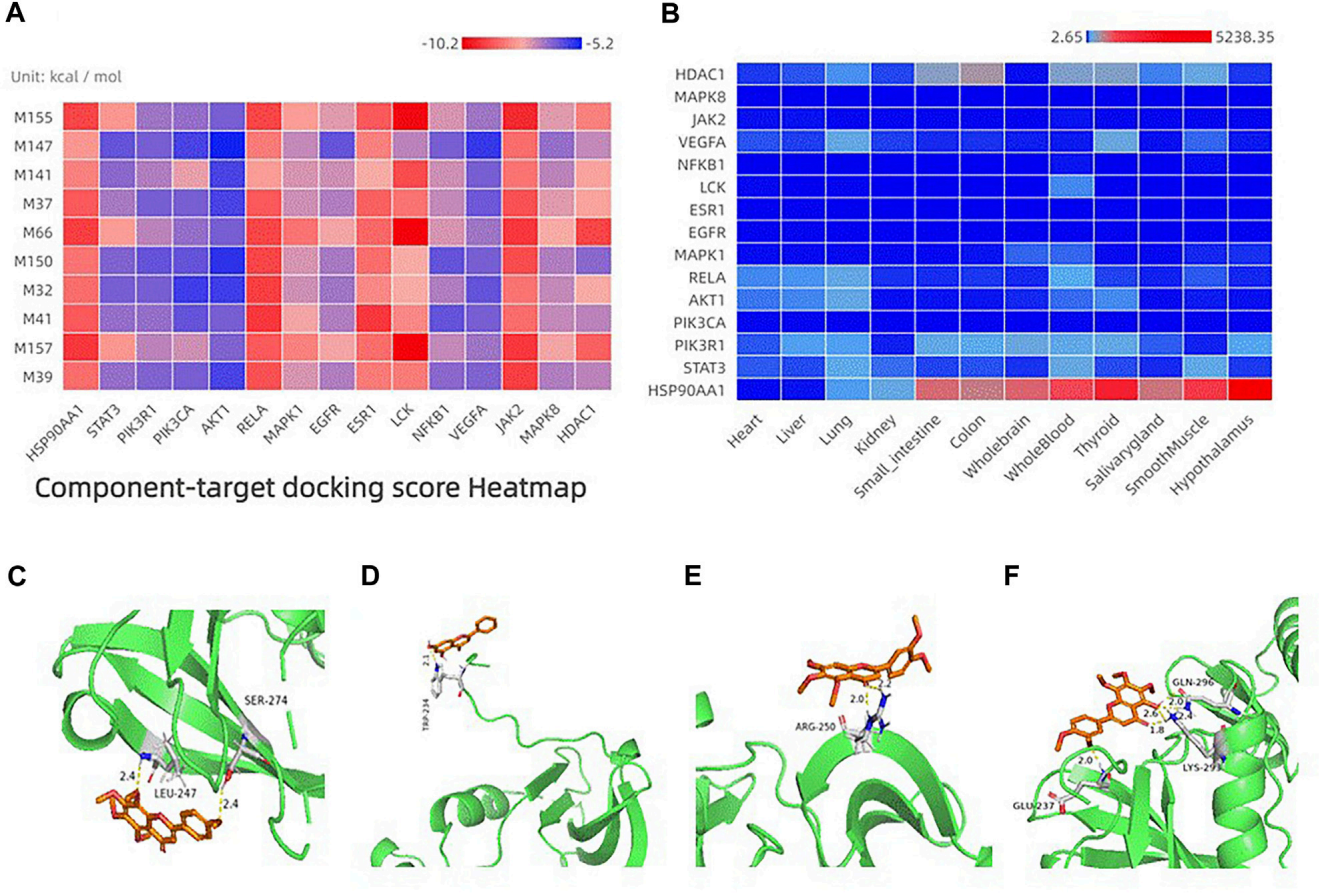
**(A)** Heatmap of the top 15 targets docking with the top 10 components. The x-axis indicates the target name. The y-axis indicates the component name; from top to bottom, acacetin, 3,3′,4′,5,6,7,8-Heptamethoxyflavone, xanthotoxol, tetramethylscutellarein, apigenin, 5,6,7,3′,4′,5′-Hexamethoxyflavone, tangeretin, 5-Demethylnobiletin, chrysin, sinensetin. **(B)** Heatmap of organ expression of 20 targets. The x-axis indicates the organ name. The y-axis indicates the target name; from left to right, heart, liver, lung, kidney, small intestine, colon, whole brain, whole blood, thyroid, salivarygland,smoothmuscle, and hypothalamus. **(C–F)** Binding studies of selected compound–target interactions. **(C)** Tangeretin with LCK. **(D)** Chrysin with LCK. **(E)** Sinensetin with LCK. **(F)** 5-demethylmobiletin with LCK. Molecules are represented by a ball-and-stick model. Hydrogen bonds are represented by a dotted line, and numbers indicate hydrogen-bond distances in angstroms.

Expression of the top-15 targets in the PPI-network according to the node degree in different organs is shown in Figure 4B (see the Supplementary Table for specific values). The redder the color, the higher is the expression of that target. Conversely, the bluer the color, the lower is the expression of that target. Most of the targets had high expression in the liver, kidneys, small intestine, lungs, and thyroid gland. According to TCM theory, pathogenesis is a complex process involving a group of interrelated factors. Therefore, in the treatment of local lesions, the mutual influence of different organs on physiology and disease should also be based on the holistic principles (Xu et al., 2021).

Study found that TCM can alleviate dry mouth in patients with head-and-neck cancer after radiotherapy. This effect is achieved by promoting the flow and secretion of salivary amylase, and protecting the salivary glands of patients (Wang et al., 1998). Some scholars analyzed the pathological characteristics of “dryness” in patients living in Hunan Province, China. They concluded that dryness and dampness were due mainly to the “child disease mother” effect in the disease process (Fan et al., 2020). According to TCM theory, drynessinjuresthe lung. The lung and spleen belong to “gold” and “Earth” in the “Five Elements Theory” of TCM. Dampness tends to trap the spleen. Wet soil is the “mother of dryness”. In addition, excessive lung dryness will hurt the spleen “soil”. Hence, the organs of the human body are interrelated and also interact with each other.

We showed that therapeutic targets could exist in two or more organs simultaneously, and that multiple targets could be distributed in the same organ. This finding suggests that human organs are related to each other during the occurrence and development of diseases, and FA may have a therapeutic role in SS-like symptoms through multiple targets and multiple pathways.

### 3.8 Computational assessment of selected compound–target interactions

In general, the lower the binding energy, the more stable the binding between the ligand and receptor. Binding energy <0 indicates that a ligand molecule can bind spontaneously to a receptor molecule (Namasivayam and Günther, 2007). Therefore, we explored the interaction and binding modes between the top-15 targets in the PPI network and their potential active components by AutoDock Vina. Through molecular docking, we can determine the possibility of potential components acting on the target and the specific binding sites. The results of molecular docking are shown in Figure 4A.

Previous analyses of compound-target networks have revealed close associations of the components sinensetin, tangeretin, 5-demethylnobiletin, and chrysin with FA targets. Therefore, we first undertook molecular docking of LCK with them. The binding energy between sinensetin and LCK was −8.4 kcal/mol, which indicated the possibility of strong binding between them. Sinensetin and LCK formed a hydrogen bond at arginine (Arg)-250 (Figure 4E). The molecular-docking results of tangeretin with LCK showed that the binding energy between them was −7.7 kcal/mol, which suggested good binding activity. Figure 4C shows the formation of hydrogen bonds at leucine (L)-247 between tangeretin and LCK. The binding energy between 5-demethylnobiletin and LCK was −8.2 kcal/mol, and the binding energy between chrysin and LCK was −10.2 kcal/mol, respectively, which indicated the possibility of strong binding between them. LCK could form hydrogen bonds with 5-demethylnobiletin at glutamic acid (Glu)-237, lysine (Lys)-293, and glutamine (Gln)-296 (Figure 4F). Moreover, LCK could form hydrogen bonds with chrysin at tryptophan (Trp)-234 (Figure 4D). Supplementary Table shows the specific values.

Based on these data, we considered that: 1) The interactions between these active ingredients and targets underlie their biological activity; 2) The drying effect of FA is reliant on multiple components and multiple targets.

### 3.9 Experimental evaluation

#### 3.9.1 Determination of dose

In the early stage of this experiment, different dose groups (1-, 10-, 20-times of the equivalent dose) were set. After 4 weeks of administration, we found that the administration effect of the four components could all reach the same level as that of FA decoction with the same dose, and the effects of some components were more significant. Comprehensive analysis of various indicators (water consumption, urine volume, rates of salivary secretion, fecal water content, aquaporin (AQP)5 expression in the kidneys, area of the long axis of the submandibular gland) revealed that a 10-times equivalent dose of each component could better reflect the administration effect than that of other dose groups. Therefore, this dose was selected for the subsequent experiment. The four initially selected validation components were not screened.

#### 3.9.2 *In Vivo* experiment

Mice in each administration group drank more water (to varying degrees) than mice in the control group. The fecal water content, total volume of urine, and salivary flow rate of mice in each administration group were reduced (to varying degrees) compared with those in the control group, and reduced further with prolongation of the administration period (see the Supplementary Table for details).

**FIGURE 5:**
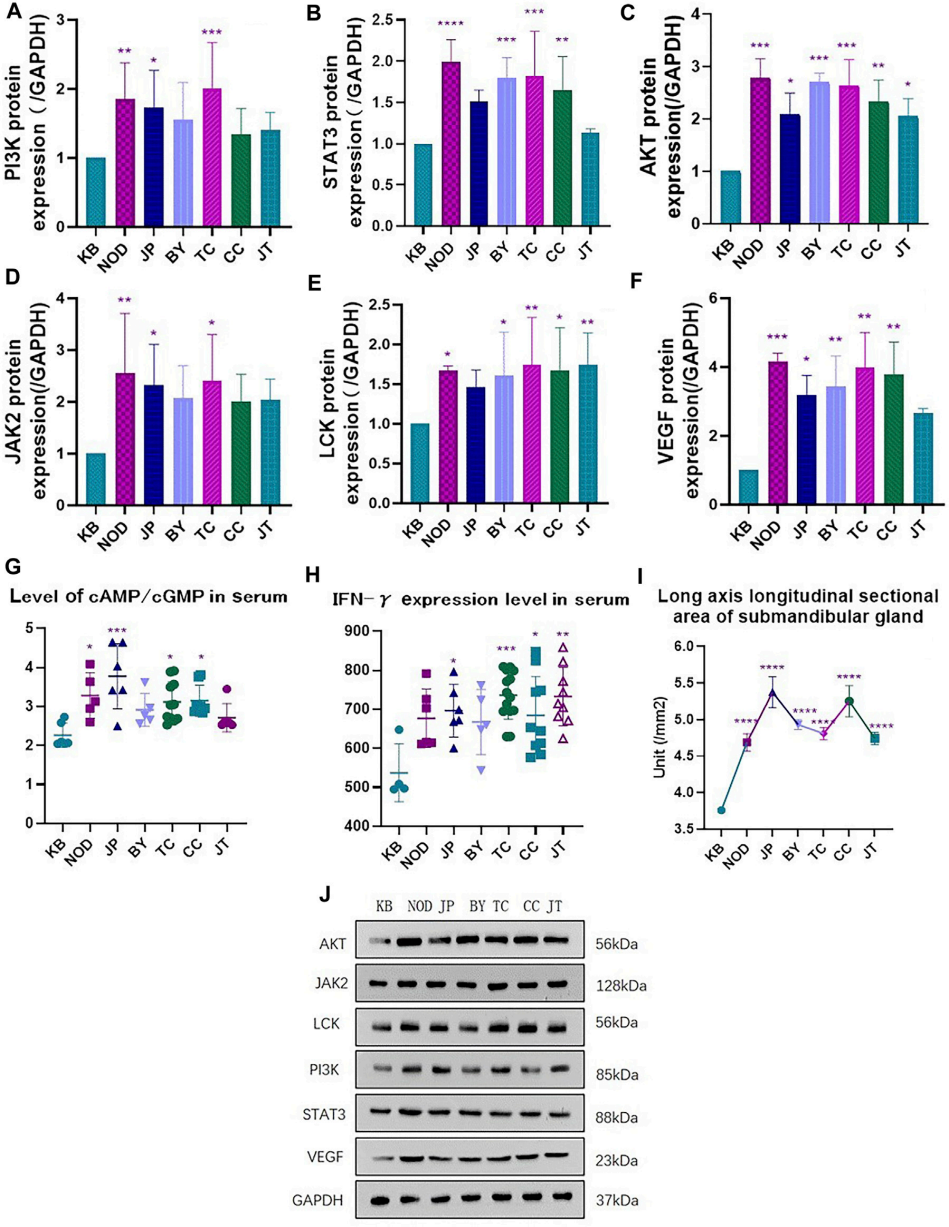
**(A)** Expression of PI3K protein. **(B)** Expression of STAT3 protein **(C)** Expression of AKT protein. **(D)** Expression of JAK2 protein. **(E)** Expression of LCK protein. **(F)** Expression of VEGF protein. **(G)** Level of cAmp/cGmp expression in mice serum. **(H)** Expression of inflammatory factors IFN-γin mice serum. **(I)** Longitudinal sectional area of submandibular gland long axis in each group. **(J)** Western blotting.*p < 0.05, **p < 0.01 vs. control group (KB).

The area of the long axis of the submandibular gland in mice in each administration group was significantly greater than that of mice in the control group (Figure 5I).

Compared with the control group, the serum level of interferon-γ and the ratio of the serum level of cAMP:cGMP in each administration group was increased (to varying degrees), among which there were significant differences in the administration groups of sinensetin, tangeretin, and 5-demethylnobiletin (Figure 5H).

The serum interferon-γ level in the extract-administration group (containing orange flavones) was significantly different from that in the control group. Also, the ratio of the serum level of cAMP:cGMP in the tangeretin group was significantly different from that in the control group. These data suggested that the ingredients stated above could cause varying degrees of body-fluid damage to healthy mice (especially tangeretin) (Figure 5G).

Western blotting showed that, compared with the control group, the extract-administration group and NOD group could increase expression of each protein to varying degrees, and each administration group showed the same upward trend. Tangeretin could upregulate protein expression of PI3K, AKT, JAK2, and VEGF significantly. Chrysin could upregulate protein expression of LCK, VEGF, STAT3, and AKT significantly. Sinensetin could upregulate protein expression of PI3K, AKT, LCK, VEGF, and STAT3 significantly. 5-Demethylnobiletin could upregulate protein expression of AKT, LCK, VEGF, and STAT3 significantly (Figures 5A–F, J).

**FIGURE 6:**
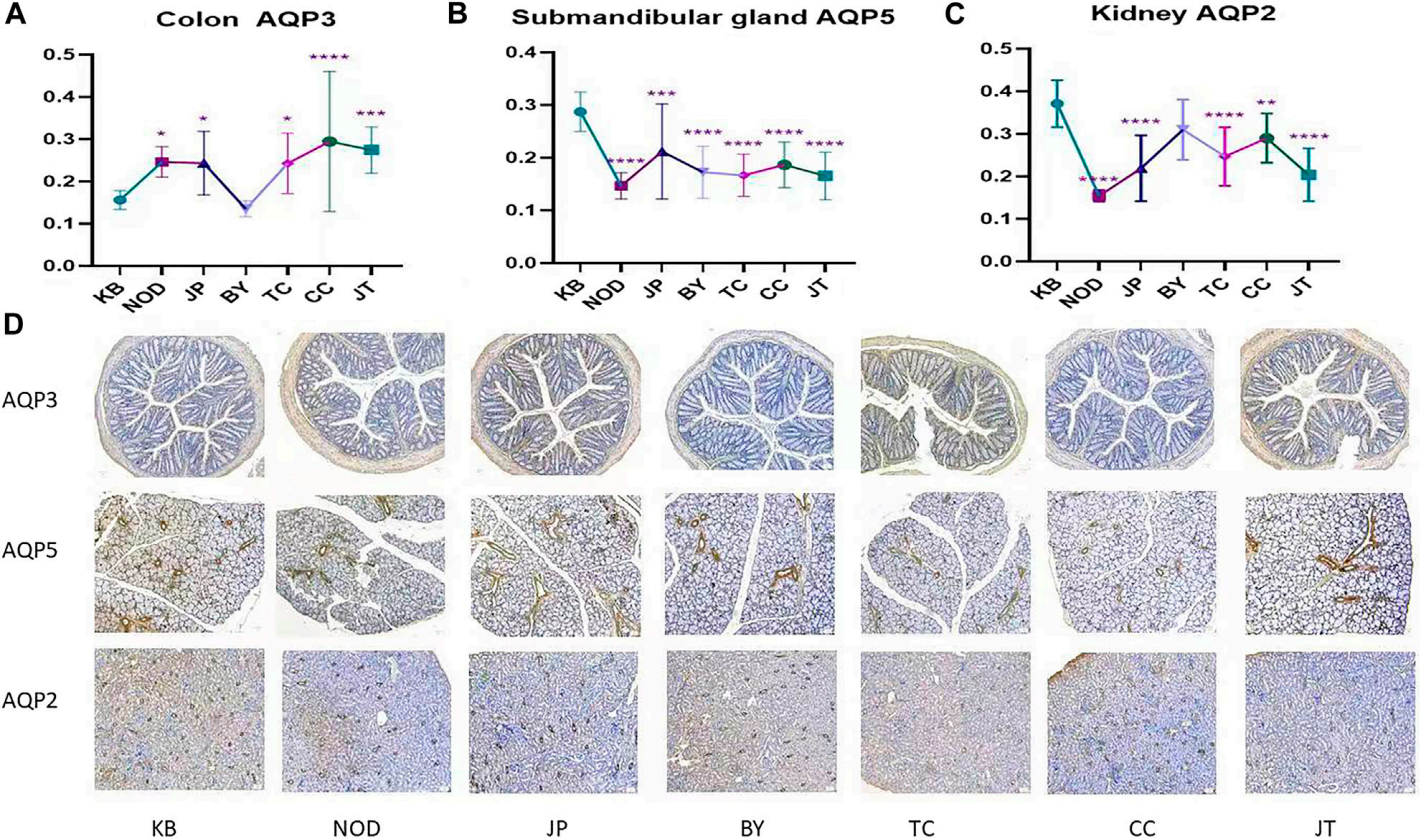
**(A)** Expression of AQP3 protein in colon. **(B)** Expression of AQP5 protein in submandibular gland. **(C)** Expression of AQP2 protein in kidney. **(D)** Immunohistochemical example diagram of each group. The y-axis indicates the protein name. The x-axis indicates the group name; from left to right, control; NOD model; tangeretin; chrysin; sinensetin; 5-demethylnobiletin; extract administration.

Immunohistochemical analysis showed that, compared with the control group, each administration group could reduce AQP5 expression in the submandibular gland significantly. Tangeretin, sinensetin, and 5-demethylnobiletin could significantly reduce AQP2 expression in the kidneys yet enhance AQP3 expression in the colon. Compared with the model (NOD) group and extract-administration group, each administration group had the same expression trend. (Figures 6A–C). The immunohistochemical sections of each administration group are shown in Figure 6D.

**FIGURE 7:**
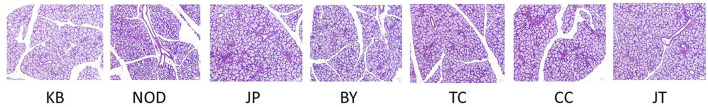
Pathological section of submandibular gland (100×). Letters in the figure indicates the group name; From left to right, control; NOD model; Tangeretin; Chrysin; Sinensetin; 5-demethylnobiletin; Extract administration.

Submandibular-gland tissues were stained with H&E and observed under light microscopy (Figure 7). In the control group, the acinus of the submandibular-gland was regular in size and closely arranged, without duct expansion or lymphocyte infiltration. In the model group, there was no obvious lymphocyte infiltration, vascular dilation,catheter-related edema in the submandibular gland, and the size of acini was different. Compared with other groups, the tangeretin group showed more lymphocyte infiltration, accompanied by acinar atrophy and deformation.

## 4 Discussion

Raw FA has a strong drying effect. If taken for a long time or inappropriately, it can cause yin-blood deficiency or yin deficiency, leading to an unmoistened nose or unmoistened throat. Initially, it manifests as xerostomia of the mouth and eyes, which accumulates over time and causes body-fluid exhaustion and blood dryness. According to TCM theory, dryness is an inherent feature of TCM formula that can lead to thirst, dry mouth, dry nose, dry skin, dry stools, and low urine output. Therefore, in the present study, body-fluid indices of mice, such as the water intake, urine output, rate of salivary secretion, and water content of feces were selected to investigate the influence of the drying effect of FA on body fluids.

Compared with the control group, the amount of drinking water of mice in each treatment group increased, and the indicators of urination, saliva secretion rate and fecal water content decreased; In addition, each test index in mice showed the same trend as that in the extract-administration group to different degrees. This result indicated that each component could have an impact on the body’s water metabolism, which preliminarily confirmed that the dryness component predicted by our model was reliable.

AQPs are proteins that can control the transmembrane transport of water with high selectivity. AQPs play an important part in the distribution, transportation, metabolism, and digestion of body fluids (Liao et al., 2021). It has been reported that different subtypes of AQP have different functions, and that the tissues they reside in are also different. AQP2 is present in the main cells of renal collecting ducts, is sensitive to antidiuretic hormone (ADH), which can regulate urine excretion (Jung and Kwon, 2016; Yu et al., 2022). Studies have shown that an increase in urine volume may be related to decreased expression of AQP2, and that protein expression of AQP2 can reflect water metabolism. Therefore, determination of AQP2 content can be used as an indicator to measure the drying effect of a TCM formulation. AQP3 is the most highly expressed AQP subtype in the colonic mucosa and has a leading role in mediating free water transport (Ikarashi, 2013). AQP5 is a commonly used marker to evaluate the drying effect of FA (Ren et al., 2020). Therefore, the contents of AQP2 in the kidneys, AQP3 in the colon, and AQP5 in the submandibular gland were selected as indicators of the drying effect of FA in our study. The metabolism of cAMP and cGMP in normal cells is in dynamic balance, and the two maintain a certain level. TCM theory holds that dryness damages yin, and patients with yin deficiency syndrome often show an increase in the cAMP level and a decrease in the cGMP level (i.e., an increase in the cAMP:cGMP ratio) (Qu et al., 2018). Studies have shown that the essence of yin deficiency and yang deficiency is closely related to the cyclic-nucleotide system. Scholars have postulated that a change in the plasma level of cyclic nucleotides in patients with yin deficiency is characterized by an increase in the cAMP level and cAMP:cGMP ratio: our results confirm this view.

The early symptoms and pathologic process of SS are very similar to the drying effect of FA (Killedar et al., 2006; Scuron et al., 2019). To further clarify the main active ingredients and molecular mechanisms of the drying effect of raw FA, we integrated network pharmacology, molecular docking, and *in vivo* experiments.

A review showed that flavonoids can be used to treat various types of tumors in clinical trials (Bisol et al., 2020). Maleki and colleagues showed that flavonoids also could inhibit the development and progression of inflammatory diseases by inhibiting the enzymes and transcription factors involved in inflammatory reactions and by acting on immune cells (Maleki et al., 2019). Recent studies have found that sinensetin can attenuate interleukin-1β-induced cartilage damage ameliorate osteoarthritis by regulating expression of serpin family A member 3, and possesses strong anticancer activities and a wide range of pharmacological (anti-inflammatory, anti-obesity, anti-dementia, vasorelaxant) activities. (Han et al., 2021; Liu et al., 2022b).

Sinensetin has been shown to inhibit activation of STAT3 phosphorylation in a phorbol 12-myristate 13-acetate plus A23187-stimulated human mast cell line in a dose-dependent manner. Activated STAT3 upregulates expression of proinflammatory mediators and induces expression of inflammation-related genes. These findings further indicate that sinensetin is involved in mast cell-mediated inflammation (Chae et al., 2017).

Wang and colleagues found that 5-demethylmobiletin regulates the JAK2-STAT3 pathway, thereby inhibiting JAK2 expression and STAT3 phosphorylation (Wang et al., 2019). Qi and collaborators discovered that chrysin can inhibit lipopolysaccharide-induced phosphorylation of JAK-STATs, nuclear translocation of STAT1 and STAT3, and release of tumor necrosis factor-α, monocyte chemoattractant protein-1, interleukin-6, and production of reactive oxygen speciesin RAW264.7 cells (Qi et al., 2018).

Mantawy and coworkers suggested that chrysin can reduce lipid peroxidation, enhance secretion of antioxidant enzymes, reduce expression of p53, Bax, Puma, Noxa, cytochrome c, caspase-3, increase expression of B-cell lymphoma-2, and inactivate mitogen-activated protein kinase, p38, and c-Jun N-terminal kinases. Chrysin can also reduce expression of nuclear factor-kappa B and phosphatase and tensin homolog, and augment the VEGF/AKT pathway. (Mantawy et al., 2014; Liang et al., 2022). *In vitro* and *in vivo*, chrysin may reduce the proliferation and induce the apoptosis and death of cells, reduce inflammation, and inhibit tumor growth by activating the Notch1 signaling pathway (Yu et al., 2012; Xue et al., 2016). Xu et al. demonstrated that tangeretin could improve allergic rhinitis by mediating inhibition of the Notch-1 signaling pathway and promoting the differentiation of regulatory T cells (Xu et al., 2019).

To sum up, we selected the four components stated above to verify the dryness effect of FA. Analyses of a PPI network revealed that the target of dryness effect was mainly due to proteases, including PIK3R1, PIK3CA, AKT1, and LCK. Enrichment analyses using the GO database showed that the drying effect of FA was mainly through acting on the endomembrane system, regulating cellular response to an endogenous stimulus, and positively regulating apoptosis, cell proliferation, and macromolecular metabolism. To a certain extent, these data also suggest that FA has multiple components, multiple targets, and multiple pathways.

## 5 Conclusion

Four components of FA (tangeretin, sinensetin, chrysin, 5-Demethylmobiletin) are closely related to the JAK-STAT3, PI3K-AKT, and the other signaling pathways. These components will be important in the drying effect of FA if it is used in a TCM formulation. They can regulate the protein expression of JAK2, STAT3, PI3K, LCK, VEGFA and AKT1, affect water metabolism in the body and, finally, result in a drying effect.

## Data Availability

The datasets presented in this study can be found in online repositories. The names of the repository/repositories and accession number(s) can be found in the article/Supplementary Material.
